# Impact of Scanbody Geometry and CAD Software on Determining 3D Implant Position

**DOI:** 10.3390/dj12040094

**Published:** 2024-04-03

**Authors:** Judith Kropfeld, Lara Berger, Werner Adler, Katja Leonie Schulz, Constantin Motel, Manfred Wichmann, Ragai Edward Matta

**Affiliations:** 1 Department of Prosthodontics, University Hospital Erlangen, Glückstrasse 11, 91054 Erlangen, Germany; judith.kropfeld@icloud.com (J.K.); lara.berger@uk-erlangen.de (L.B.); constantin.motel@uk-erlangen.de (C.M.); claudia.ehrhardt@uk-erlangen.de (M.W.); 2 Institute of Medical Informatics, Biometry and Epidemiology (IMBE) of the Friedrich-Alexander-University, Erlangen-Nuremberg, Waldstrasse 6, 91054 Erlangen, Germany; werner.adler@fau.de; 3 Department of Oral and Cranio-Maxillofacial Surgery, University Hospital Erlangen, Glückstrasse 11, 91054 Erlangen, Germany; katja.schulz@uk-erlangen.de

**Keywords:** Computer-Aided Design/Computer-Aided Manufacturing (CAD/CAM), digital workflow accuracy, implant dentistry, intraoral scanning (IOS), scanbody geometry

## Abstract

The implementation of CAD software in the digital production of implant prosthetics stands as a pivotal aspect of clinical dentistry, necessitating high precision in the alignment of implant scanbodies. This study investigates the influence of scanbody geometry and the method of superimposing in CAD software when determining 3D implant position. A standardized titanium model with three bone-level implants was digitized to create reference STL files, and 10 intraoral scans were performed on Medentika and NT-Trading scanbodies. To determine implant position, the generated STL files were imported into the Exocad CAD software and superimposed—automatically and manually—with the scanbody geometries stored within the software’s shape library. Position accuracy was determined by a comparison of the 3D-defined scanbody points from the STL matching files with those from the reference STL files. The R statistical software was used for the evaluation of the data. In addition, mixed linear models and a significance level of 0.05 were applied to calculate the *p*-values. The manual overlay method was significantly more accurate than the automatic overlays for both scanbody types. The Medentika scanbodies showed slightly superior precision compared to the NT-Trading scanbodies. Both scanbody geometry and the type of alignment in the CAD software significantly affect digital workflow accuracy. Manual verification and adjustment of the automatic alignment process are essential for precise implant positioning.

## 1. Introduction

The use of dental implants to replace missing teeth has become increasingly important in recent years and is well established in everyday clinical practice [1]. Thus, it is possible to sufficiently treat single-tooth gaps as well as more extensive gap situations and even completely edentulous jaws. Dental implants offer many advantages, as they are not only a conserving but also an aesthetic treatment alternative to conventional restorations [2]. When restoring edentulous jaws, implant-supported or -borne solutions, which are the only alternative to conventional full dentures, can provide the patient with improved functionality and comfort [3].

The clinical success of implant-supported or implant-borne restorations depends on the accuracy of the individual work steps within a coordinated work process that spans the impression, the insertion, and the finished restoration [4]. The conventional workflow begins with a precise impression, which is used to fabricate a physical plaster model in the dental laboratory. Various studies have analyzed the precision of different common impression techniques in detail and concluded that an open technique is superior to a closed impression method [5,6]. Once the model has been fabricated, the subsequent workflow can be carried out in both conventional and digital forms, with the latter involving digitizing the corresponding model using a desktop scanner. The digital workflow, on the other hand, starts with an intraoral scan (IOS) of the patient, which is immediately available for further digital processing. In addition to decreasing treatment time and increasing patient comfort, this allows immediate monitoring of the process, which can minimize potential sources of error by reducing individual manual fabrication steps [1,7,8]. IOSs have been repeatedly proven to have sufficient precision for single-tooth and short-span bridge restorations, but clinically unacceptable inaccuracies occur during the scanning process of larger gap situations or edentulous jaws [9,10,11].

The material and geometry of the scanbodies play a fundamental role in their digital acquisition since their length, width, shape, and surface quality can significantly affect the matching process, resulting in the registration of incorrect implant positions [12,13,14]. The optimization of digital impressions is therefore a current focus of both in vivo and in vitro studies, allowing the observation of further developments and advances in scanning technologies and so-called scanning aids. This enhances the precision of the IOS results, leading to the gradual replacement of conventional impressions, even in more complex intraoral situations [15,16,17,18,19].

However, the success of the treatment depends not only on the accuracy of the scan, needed to create a three-dimensional virtual model as the basis for the design, but also on its further processing using Computer-Aided Design/Computer-Aided Manufacturing (CAD/CAM) software and, ultimately, on the final mechanical and manual fabrication process of the respective restoration. Here, too, the individual work steps must be precisely coordinated; for example, the indication, material selection, design, milling strategy, and milling machine must be harmonized [20,21]. In this respect, one of the most important steps in implant restorations is the precise alignment of the scanbody mesh stored in the CAD software’s shape library with the actual scanbody digitized by the IOS. This process is essential for virtually determining the position of the implant within the CAD software and provides the basis for further digital processes in implant prosthetics [22]. The approach to the matching process varies across CAD systems. After selecting the relevant implant system, the corresponding scanbody geometry mesh from the software library is applied. An automatic alignment via a best-fit algorithm is initiated after pre-selecting either one or three points on the scanbody, depending on the specific CAD software. It is essential for the user to carefully verify the alignment of the library scanbody mesh and the scanbody in the IOS and adjust the superimposition manually if required. Adjustments may require varying the positions of the selected points or changing the considered length of the scanbody for optimal alignment accuracy. The risk of clinically unacceptable results increases considerably if this work step is insufficiently accurate, resulting in incorrect implant positions [10,19]. Regarding general digitalization in dentistry, many digital work steps have already been extensively investigated to increase speed and comfort during patient treatment and to improve the quality and efficiency of dental restorations. However, most research has focused exclusively on the accuracy of the final restoration, with only a few studies focusing on the specific technical steps within the CAD/CAM software. Thus, an understanding of the challenges of the software components has often been neglected.

In response, this study analyzes the automatic and manual alignment processes of two different scanbodies in the Exocad CAD software (v3.0; Exocad GmbH, Darmstadt, Germany; Dental CAD Rijeka) to identify potential weaknesses and further improve the accuracy of the digital workflow and thus the quality of implant-supported and -borne dental prosthetics.

The null hypothesis is that neither the CAD software nor the geometry of the scanbodies has an influence on the accuracy of the matching process.

## 2. Materials and Methods

The objective of the in vitro study carried out at the Dental Clinic 2—Dental Prosthetics of the University Hospital Erlangen was to investigate the accuracy of implant position in Exocad software (Exocad GmbH, Darmstadt, Germany; Dental CAD Rijeka) depending on (1) the superimposition method of the scanbodies and (2) the use of two different scanbody shapes.

**Figure 1:**
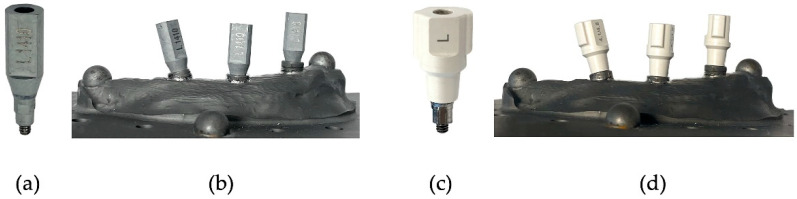
(**a**,**b**) Medentika scanbodies: separate and screwed onto the reference titanium model, respectively. (**c**,**d**) NT-Trading scanbodies: separate and screwed onto the reference titanium model, respectively.

Scanbodies from the companies Medentika (L-Series L1410; Straumann GmbH, Freiburg im Breisgau, Germany) and NT-Trading (L-Series L 9.S3D4.148; NT-Trading GmbH & Co. KG., Karlsruhe, Germany) were selected in advance for the proposed investigations (see Figure 1a,c). Following the manufacturer’s recommendation, the scanbodies were manually tightened by hand to prevent excessive force that could be applied using a torque wrench, ensuring proper installation.

Furthermore, a reference model of an edentulous maxilla was milled from titanium using computerized numerical control (CNC) for a standardized experimental setup. Three bone-level implants (TiZr, ø 4.8 mm RC, SLA 12 mm, Roxolid, Loxim; Straumann AG, Basel, Switzerland) were then placed in this model. Of these, two implants in the frontal and premolar regions were inserted perpendicularly to the model base, and the third was angulated 15 degrees distally in the molar region. The positioning and orientation of the implants within the model were based on a real patient case in order to simulate a clinical practice scenario. These implants were precisely placed within the titanium model; however, their inability to undergo osseointegration for stabilization—akin to their function in bone—necessitated an alternative approach. In collaboration with the implant manufacturer, we opted for laser welding to ensure their firm attachment. This process involved using a dental laser to create a weld seam both around the implant’s exterior and at the drill stud’s edge in the titanium model, ensuring the preservation of the integrity of the implant’s interface.

**Figure 2:**
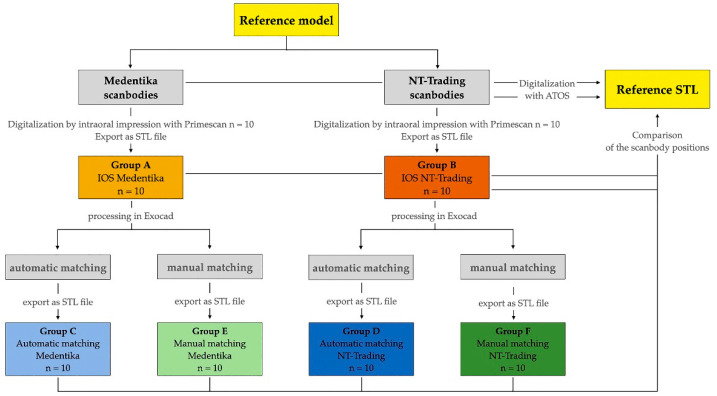
Representation of the study procedure with the individual study groups.

As described in the following section, Figure 2 provides an overview of the study design and the individual study groups.

To generate a reference file and thus compare scanbody and, consequently, implant positions, the reference model was digitized with respective scanbodies from Medentika and NT-Trading. This process was carried out using the high-precision ATOS So4 II (GOM GmbH, Braunschweig, Germany) industrial white-light scanner. As a result, one reference Standard Transformation Language (STL) file was created for the Medentika scanbodies and another for the NT-Trading scanbodies (see Figure 1b,d).

Subsequently, a procedure was utilized as it would be performed on a patient to stay as close to clinical conditions as possible despite the in vitro study setup. The titanium model was equipped with three scanbodies from one manufacturer and digitized using the Primescan intraoral scanner (Dentsply Sirona, Bensheim, Germany; Primescan AC; Connect SW 5.2). Then, the scanbodies were unscrewed and re-screwed for the next scan, incorporating this potential source of error that could occur with multiple uses of a scanbody on a patient. This process was repeated 10 times each for both Medentika and NT-Trading, and the scans were exported as STL files.

The data sets were then imported individually into Exocad for further processing, which allowed both automatic and manual determination of the implant position. In the automatic procedure, a scanbody stored in the software can be overlaid three-dimensionally with the scanbody of the IOS STL, using one-point alignment over a specified position in the center of the scanbody. The software then applies an automatic best-fit algorithm for an optimal matching result. This process was carried out individually for each IOS STL, and the resulting data sets were exported as new STL matching (automatic) files.

Subsequently, implant position was recorded using the manual overlay method. The original IOS STL files were re-imported to avoid manipulation of the previously recorded data by means of automatic alignment. In contrast to automatic matching, in addition to placing the alignment point in the center of the scanbodies, the observer changed position repeatedly until determining that the software calculated an exact superimposition of the scanbodies after the best-fit process. A color visualization of the deviations enabled an additional inspection of the accuracy of the respective superimposition process. After this process, all matched virtual models were also saved and exported as STL matching (manual) files.

The accuracy of the scanbody position within the various STL matching (automatic/manual) data sets was analyzed by superimposing the respective reference STL files in ATOS Professional software (v2018; GOM GmbH, Braunschweig, Deutschland) using a best-fit alignment of the surface without the scanbodies. To exclude the intraoral scanning process as a possible source of error, the IOS STLs were also examined by matching them with the reference files. During this process, all IOS scans (*n* = 20) and all STL files generated from Exocad (*n* = 40) were divided into groups and compared with the respective reference STLs (Medentika or NT-Trading). Groups A and B represent the IOS STLs with the Medentika and NT-Trading scanbodies, respectively. Groups C and D correspond to the Exocad STL matching (automatic) files generated through automatic matching with the Medentika and NT-Trading scanbodies, respectively. Groups E and F represent the manual superimposition for STL matching (manual) files for the Medentika and NT-Trading scanbodies, respectively.

**Figure 3:**
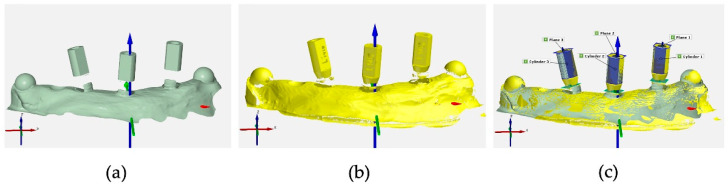
Example of the virtual calculation of 3D deviations in ATOS Professional: (**a**) Exocad-generated STL after the manual matching process, (**b**) reference STL, and (**c**) superimposition of the reference STL with the Exocad-generated STL using fitting cylinders and planes.

To specifically determine the deviations in the recorded scanbody positions, defined points were calculated on each scanbody on both the reference and the IOS and Exocad-generated STLs to be examined. Their position in relation to each other was determined in a coordinate system defined as follows: the *x*-axis represented the mesio-distal direction, the *y*-axis represented the oro-vestibular direction, and the *z*-axis represented the vertical direction. To determine the position of the scanbody in the x- and y-directions, a fitting cylinder having maximum contact with the partly round outer surfaces of the scanbodies was placed around each scanbody. A maximum congruent fitting plane was generated on the top of the scanbody to record the localization along the *z*-axis. The point to be compared in the coordinate system was found at the intersection of the cylinder axis with the plane, which was calculated for both reference STLs as well as all IOS STLs (Groups A and B) and Exocad-generated STLs (Groups C, D, E, and F). In addition, the Euclidean distance dXYZ was calculated as a 3D deviation resulting from the vector of the displacement from the three axes. The discrepancy in the corresponding points was determined using ATOS Professional software (GOM GmbH) in the form of mean, maximum, and minimum deviations in mm (see Figure 3). Similar evaluation methods have been used successfully in other studies and can therefore be classified as suitable [5,12,23].

The statistical analysis of the collected data was performed with the R program (R Core Team, R Foundation for Statistical Computing, Vienna, Austria) [24]. Mixed linear models were used to calculate the *p*-values, and the significance level was set at 0.05.

## 3. Results

Overall, the data sets obtained indicate that the IOS STLs of Groups A and B (Medentika and NT-Trading) have very small deviations from the initially generated reference STL files, averaging 22 ± 11 µm and 31 ± 8 µm, respectively, in the dXYZ direction.

**Figure 4:**
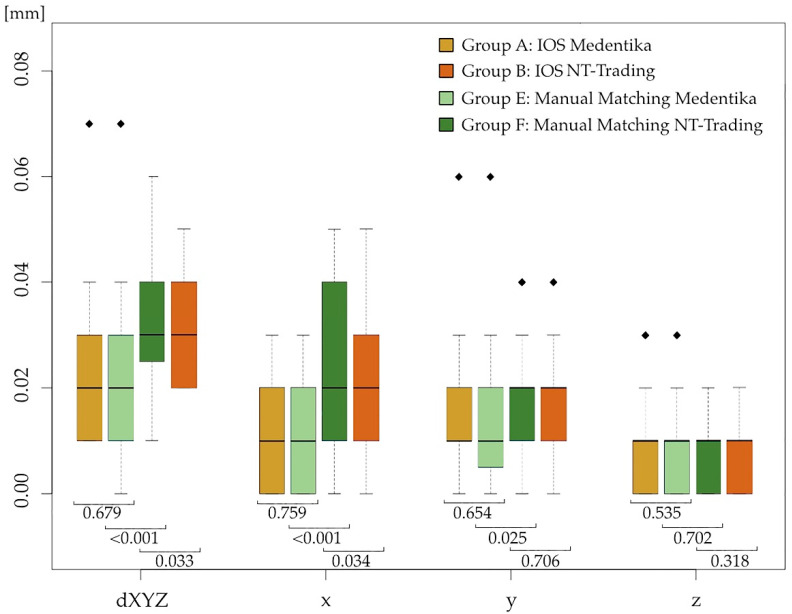
Statistical comparison of the scanbodies’ deviations im mm of Groups A (IOS Medentika) and E (Manual Matching Medentika), E (Manual Matching Medentika) and F (Manual Matching NT-Trading), and F (Manual Matching NT-Trading) and B (IOS NT-Trading), with corresponding p-values for the Euclidean distance dXYZ and the x-, y-, and z-directions.

**Figure 5:**
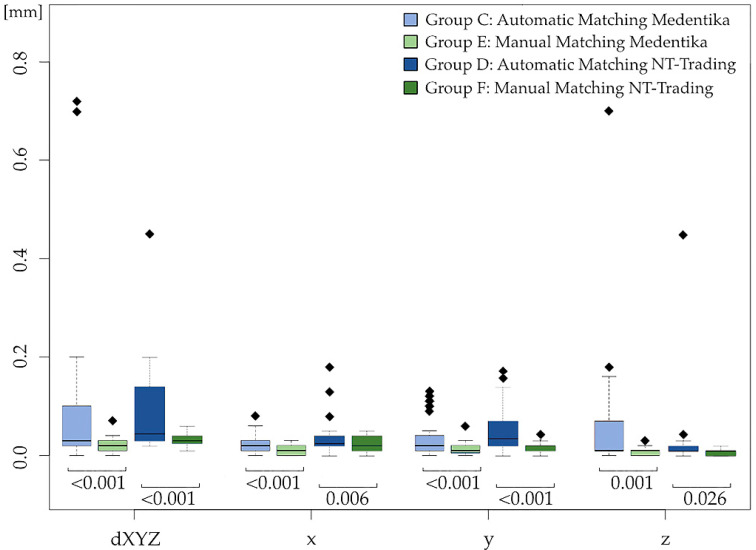
Boxplots with *p*-values for the comparison of the scanbodies’ deviations with automatic and manual overlay methods for Medentika and NT-Trading: Group C (Automatic Matching Medentika) vs. E (Manual Matching Medentika), Group D (Automatic Matching NT-Trading) vs. F (Manual Matching NT-Trading)) for all directions (dXYZ, x, y, z).

With regard to the implant position calculated using Exocad, in the dXYZ direction, an average deviation from the reference STL of 84 ± 132 µm (C) was recorded for Medentika in the automatic matching procedure and of 21 ± 11 µm (E) in the manual matching process, while slightly lower values of 94 ± 103 µm (D, automatic matching) and 35 ± 13 µm (F, manual matching) were recorded for NT-Trading. Detailed information on the group-specific deviations along the *x*-, *y*-, and *z*-axes is displayed in Table 1 and Figure 4 and Figure 5.

Regarding the superimposition process, significantly higher accuracy was found for both Medentika and NT-Trading for the manual overlay option (Groups E, F) compared to the automatic method (Groups C, D) (*p* < 0.001). In the automatic overlay process, the Medentika scanbodies exhibited the largest deviations in the *z*-axis at 54 ± 103 µm (C), whereas this axis showed the smallest deviation, 8 ± 7 µm, after manual processing (E) (*p* = 0.001). In contrast, the greatest deviations after automatic overlaying of the NT-Trading scanbodies (D) were found in the *x*-axis (45 ± 54 µm) and *y*-axis (49 ± 43 µm). Along the *z*-axis, however, the deviations for the NT-Trading scanbodies were significantly smaller for both automatic (34 ± 97 µm, D) and manual (7 ± 7 µm, F) processing (*p* = 0.026).

Considering the different scanbodies of Medentika and NT-Trading, a statistical comparison indicates a significant slightly higher accuracy on average for the Medentika scanbodies (E) in the manual overlay process compared to NT-Trading (F) in the dXYZ direction (*p* < 0.001), in the mesio-distal dimension (*x*-axis, *p* < 0.001), and along the oro-vestibular axis (*y*-axis, *p* = 0.025).

## 4. Discussion

In recent years, digitization in dentistry has become an increasingly important and integral component of contemporary treatment methodologies. Specifically, the utilization of CAD/CAM systems in implant prosthodontics represents a significant advancement, potentially enhancing both the quality of treatment and the efficiency of therapeutic care options. [11,20,25]. The final accuracy of the restoration fit depends on a wide range of factors, and inaccuracies in IOSs have been described in the literature as a possible source of error in the digital workflow [10,15,26,27]. However, numerous other factors can also significantly impact the accuracy of IOSs. These include the technical skills of the practitioner [9,22], external influences such as lighting conditions [11,28], complex oral circumstances (implant position, blood and saliva flow, soft tissues, adjacent teeth) [9], and the material and geometry of the scanbody [9,12,22]. In addition to these aspects, planning and design, the skills of the dental technician, and CAD/CAM software solutions also play a significant role across the digital workflow and need to be further investigated. [22].

In this context, the present study focuses on a crucial aspect of the digital workflow: the accuracy of implant positioning depending on the alignment method of Exocad and the geometry of different scanbodies (Medentika, NT-Trading). Overall, the initial null hypothesis can be rejected, as the manual superimposition process was associated with higher accuracy for both scanbody shapes compared to the automatic method. Furthermore, the Medentika scanbodies showed slightly higher precision compared to the NT-Trading scanbodies.

The obtained results show the high quality of the originally created IOS of both Groups A and B due to small deviations at a maximum of 31 ± 8 µm from the two reference data sets. Related to current studies on impression methods [5,11,27], this can be assessed as being very accurate and allows the control of a potential measuring bias. At this point, however, it is important to note that the IOS impressions in this study were conducted under in vitro conditions, which are ideal but may not accurately reflect in vivo conditions where various influencing factors can lead to greater inaccuracies. Nonetheless, within the scope of this study, which focuses on the accuracy of 3D implant positioning using software, the process of intraoral scanning did not bias the comparison of the different scanbodies. Furthermore, almost no deviation in the *x*-, *y*-, and *z*-axes of the intraoral scans (Groups A and B) compared to the manually corrected scanbody position (Groups E and F) was observed (see Table 1 and Figure 2). This indicates that the accuracy of scanbody positioning in digital workflows is more affected by the intraoral impression-taking process itself than by manual adjustments in the CAD software, emphasizing the critical importance of selecting the right IOS to minimize scanning bias and avoid subsequent inaccuracies.

To test the null hypothesis, the deviation of the different scanbody shapes along the Euclidean distance and the *x*-, *y*-, and *z*-axes compared to the two reference data sets was examined. The slightly higher accuracy of the Medentika scanbodies relative to the NT-Trading scanbodies can be attributed to the different design and material characteristics of the scanbodies [9,12,22].

**Figure 6:**
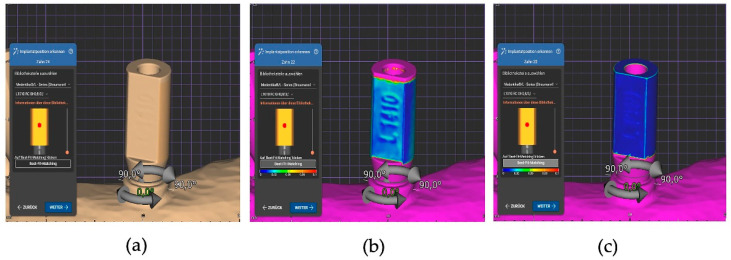
Alignment process of a Medentika scanbody in Exocad: (**a**) automatic matching with deviation in the *z*-axis, (**b**) false-color visualization of the automatic matching with deviation in the *z*-axis, and (**c**) false-color visualization of the manually corrected scanbody alignment.

The notable vertical deviations of 54 ± 103 µm (C) (*z*-axis) observed with the Medentika scanbodies during the automatic matching process could be attributed to the cylindrical shape of these scanbodies; as in the automatic one-point best-fit matching process, both the set points and the largest possible surface area should be superimposed. This is also the case with a slight offset in the *z*-axis, as the entire outer surface of the cylinder is superimposed even after parallel displacement and makes up the largest part of the scanbody. This error could be corrected through manual overlay and verification. Large discrepancies in this direction can lead to incorrect occlusion and an inadequate abutment–implant connection [29]. In contrast, the *x*- and *y*-axes were not affected by the cylindrical geometry of the Medentika scanbody and offered very accurate results in the automatic superimposition (see Figure 6).

**Figure 7:**
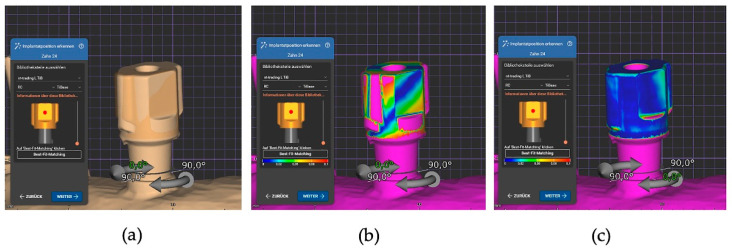
Alignment process of an NT-Trading scanbody in Exocad: (**a**) automatic matching with rotation around the *z*-axis; (**b**) false-color visualization of the automatic matching with rotation around the *z*-axis; (**c**) false-color visualization of the manually corrected scanbody alignment.

In the automatic matching process of the NT-Trading scanbodies, the greatest deviations were observed in the mesio-distal *x*-axis at 45 ± 54 µm and the oro-vestibular *y*-axis at 49 ± 43 µm (D). These can clinically lead to dislocations of the approximal and occlusal contacts. Due to the more complex geometry of these scanbodies, errors such as a parallel displacement in the *z*-axis can be practically eliminated. However, with the one-point selection, close attention must be paid to the selection of the two lateral surfaces of the scanbody to avoid a rotation of 180° around the *z*-axis (see Figure 7).

This shows that there are varying deviations in different directions for both scanbody geometries, meaning that scanbody geometry has an impact on the accuracy of the matching process. This observation is consistent with other studies [22,30,31] and emphasizes the need for continuous development and optimization of scanbody technology.

In further analyses, the deviations with automatic and manual overlays were compared. The significantly smaller deviation from the reference STL using the manual method for both scanbody types compared to the automatic overlay highlights the importance of manual adjustment in the CAD software (v 3.0). The accuracy of the automatic overlay of dental CAD systems depends significantly on the initial alignment, which is based on either a manual one-point or three-point pre-alignment approach. The different placements of these reference points result in different positionings for the scanbody. As the best-fit algorithm used in the CAD software and its exact parameters are not known, it is currently not possible to draw a definitive conclusion on this aspect. Nevertheless, this suggests that, despite the advancement of automation in digital workflows, careful manual checking and correction are essential to achieving accurate results. This observation is consistent with the findings of Gracis et al. [22], which highlighted the limitations of automated processes in digital workflows.

The present investigations have certain limitations that must be considered in the interpretation of the results. One factor results from the previously mentioned in vitro design of this study. The titanium model only applies to one possible intraoral situation, which can differ significantly among patients, leading to possible variations in implant positioning. Such variations can subsequently affect the scanning results, thereby affecting the entire digital workflow process. 

In addition, the model digitization was performed under idealized conditions that simplified a complete and error-free acquisition of the scanbodies for the practitioner. More complex oral conditions in a patient may limit the comparability of this study with clinical applications.

Furthermore, in this study, only one intraoral scanner (Primescan) was used in combination with one specific CAD software platform (Exocad). Future research should investigate how different IOS and CAD software solutions affect certain aspects of the digital workflow, such as the compatibility of different IOS systems with the software, which might have a relevant influence on the results. In addition, the matching procedures for different software platforms differ. For example, Exocad uses a one-point alignment, while other software solutions use a three-point alignment, which may potentially lead to deviations in the accuracy of the best-fit alignment and the overall matching process.

Hence, the results of this study are specifically applicable to the Primescan IOSs, the Exocad CAD software, and the scanbodies used from Medentika and NT-Trading. Future studies should therefore focus on investigating the transferability of these results to clinical practice and extending their applicability to different scanbody geometries and software solutions in order to increase the general validity of the results.

Nevertheless, the present study highlights the importance of precise superimposition of scanbodies in CAD software depending on their geometry in the fabrication of implant-supported or implant-borne restorations to guarantee accurate fitting for the patient and long-term treatment solutions using implant prosthetics.

## 5. Conclusions

Within the limitations of this in vitro study, the following conclusions were drawn:

1. The type of superimposition process within the CAD software (automatic/manually corrected) has an influence on the accuracy of the virtual model.
2. Scanbody geometry can play an important role in data acquisition and overlay in the CAD software.
3. A manual verification of the automatic superposition is essential and reduces the effects of scanbody geometry.

Based on this study, verification and manual adjustment of the automatic overlay process are recommended.

## Figures and Tables

**Table 1:** Display of the mean distance with standard deviation (SD) and the minimum (Min) and maximum (Max) distance in µm between the STLs of the respective groups (A–F) and the corresponding reference files for the *x*-, *y*-, and *z*-axes as well as the Euclidean distance dXYZ.

Group			Mean	SD	Min	Max
Group A	IOS Medentika	dXYZ	22	11	10	70
		x	12	10	0	30
		y	13	10	0	60
		z	7	8	0	30
Group B	IOS NT-Trading	dXYZ	31	8	20	50
		x	19	12	0	50
		y	17	10	0	40
		z	6	6	0	20
Group C	Automatic matching Medentika	dXYZ	84	132	0	720
		x	22	20	0	80
		y	29	33	0	130
		z	54	103	0	700
Group D	Automatic matching NT-Trading	dXYZ	94	103	20	450
		x	45	54	0	180
		y	49	43	0	170
		z	34	97	0	45
Group E	Manual matching Medentika	dXYZ	21	11	0	70
		x	11	10	0	30
		y	12	10	0	60
		z	8	7	0	30
Group F	Manual matching NT-Trading	dXYZ	35	13	10	60
		x	25	16	0	50
		y	16	11	0	40
		z	7	7	0	20

## Data Availability

The data sets used and/or analyzed during the current study are available from the corresponding author upon reasonable request.

## List of Abbreviations

CAD	Computer-Aided Design
CAM	Computer-Aided Manufacturing
3D	three-dimensional
STL	Standard Transformation Language
IOS	intraoral scan/intraoral scanner
CNC	computerized numerical control
Mean	mean distance
SD	standard deviation
Min	minimum distance
Max	maximum distance
